# Fracture mechanics by three-dimensional crack-tip synchrotron X-ray microscopy

**DOI:** 10.1098/rsta.2013.0157

**Published:** 2015-03-06

**Authors:** P. J. Withers

**Affiliations:** 1 Manchester X-ray Imaging Facility, School of Materials, Manchester University, Manchester M13 9PL, UK; 2 Research Complex at Harwell, Rutherford Appleton Laboratory, Harwell Oxford, Didcot OX11 0FA, UK

**Keywords:** computer tomography, X-ray microscopy, fracture mechanics (LEFM), synchrotron X-rays

## Abstract

To better understand the relationship between the nucleation and growth of defects and the local stresses and phase changes that cause them, we need both imaging and stress mapping. Here, we explore how this can be achieved by bringing together synchrotron X-ray diffraction and tomographic imaging. Conventionally, these are undertaken on separate synchrotron beamlines; however, instruments capable of both imaging and diffraction are beginning to emerge, such as ID15 at the European Synchrotron Radiation Facility and JEEP at the Diamond Light Source. This review explores the concept of three-dimensional crack-tip X-ray microscopy, bringing them together to probe the crack-tip behaviour under realistic environmental and loading conditions and to extract quantitative fracture mechanics information about the local crack-tip environment. X-ray diffraction provides information about the crack-tip stress field, phase transformations, plastic zone and crack-face tractions and forces. Time-lapse CT, besides providing information about the three-dimensional nature of the crack and its local growth rate, can also provide information as to the activation of extrinsic toughening mechanisms such as crack deflection, crack-tip zone shielding, crack bridging and crack closure. It is shown how crack-tip microscopy allows a quantitative measure of the crack-tip driving force via the stress intensity factor or the crack-tip opening displacement. Finally, further opportunities for synchrotron X-ray microscopy are explored.

## 1. Introduction

Conventional linear elastic fracture mechanics (LEFM) provides an established basis for analysing subcritical crack growth in terms of parameters (e.g. stress intensity factor *K*, crack-tip opening displacement (CTOD) *J*) that capture the local conditions at the crack tip, yet can be determined solely in terms of the crack length, externally applied loading and geometrical parameters. This global approach has served us very well, even in the case of relatively inhomogeneous materials, provided the crack is long relative to the scale of the heterogeneity. However, it neglects the underlying competition between the intrinsic damage mechanisms occurring ahead of the crack and the extrinsic shielding mechanisms occurring behind the crack tip that influence fatigue behaviour [1]. It is these micromechanisms that lie at the heart of the step-jumps in subcritical crack growth resistance achieved in new materials by tailored heterogeneity and interfaces at the micro- and nanoscales [2,3]. These mechanisms can be exploited in materials design to retard crack growth via repair agents [4], residual stresses [5,6], phase transformations [7], plasticity-induced closure [8], crack deflection [9] and crack bridging [10], etc. In such cases, while fracture mechanics can still be used as the principal means of predicting crack growth, traditional external indicators such as applied load and crack length are not sufficient, because processes near the crack tip modify the effective crack-tip driving force (i.e. *K*^eff^≠*K*^applied^).

**Figure 1.**
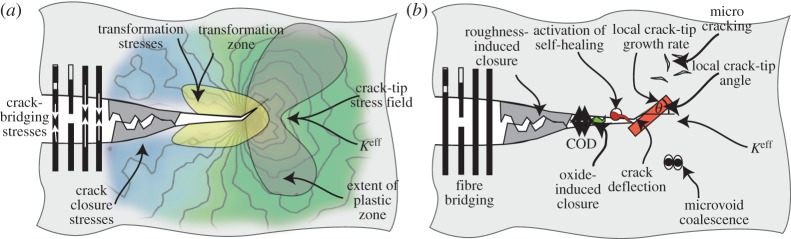
Schematic illustrating the wide range of qualitative and quantitative fracture mechanics information that can be obtained by (*a*) diffraction and (*b*) imaging [11]. Of course, not all the shielding mechanisms shown here are likely to be available in any particular case. (Online version in colour.)

To progress, we need a means of extracting quantitative information about the local crack-tip environment. Emerging techniques such as digital image correlation allow the crack-tip deformation field to be probed at the surface, but many cracks are either inherently three dimensional or initiate in the interior. This review describes the concept of three-dimensional X-ray microscopy, combining diffraction and imaging modes as in a transmission electron microscope to probe the conditions at the crack tip (figure 1). Instruments capable of this are beginning to emerge, such as ID15 at the European Synchrotron Radiation Facility (ESRF) and JEEP at the Diamond Light Source. This review explores the types of information that can be provided by diffraction (figure 1*a*) and by CT imaging (figure 1*b*). For reasons of space, many practical aspects associated with residual stress mapping by diffraction and imaging by computer tomography are not covered in this paper, but excellent introductions to both techniques are available; for example, [12] and [13,14] for diffraction and imaging, respectively.

Synchrotron X-ray microscopy provides a means of shedding new light on crack-tip phenomena allowing the efficacy of micro- and nanostructural tricks currently being exploited by the materials designer (and nature) to be quantified, thereby opening up new avenues to design the novel failure-resistant materials of the future. In this paper, the complementary insights provided by diffraction (in §2) and imaging (in §3 and §4) modes are explored across a range of materials and applications with a particular emphasis (in §5) on obtaining quantitative measures of the crack driving force before considering wider applications of X-ray microscopy.

## 2. Diffraction from the crack-tip region

In the following subsections, the types of information that diffraction can provide are investigated through a series of examples. Through the use of apertures, it is possible to ensure that the diffracted signal comes from just a small ‘gauge’ volume. This enables mapping in three dimensions. Often such measurements are focused on a region of interest, for example the volume or area local to a crack tip or defect; often, it is sufficient for the mapping to be undertaken over a two-dimensional slice, for example at the mid-plane of the sample (see §2*a*), or along a line, for example along the centre line of a crack plane (see §2*d*(i)). Some methods such as diffraction contrast tomography (see §3*b* and also §4*c*) are inherently three dimensional in nature.

### (a) Mapping crack-tip stress fields

**Figure 2.**
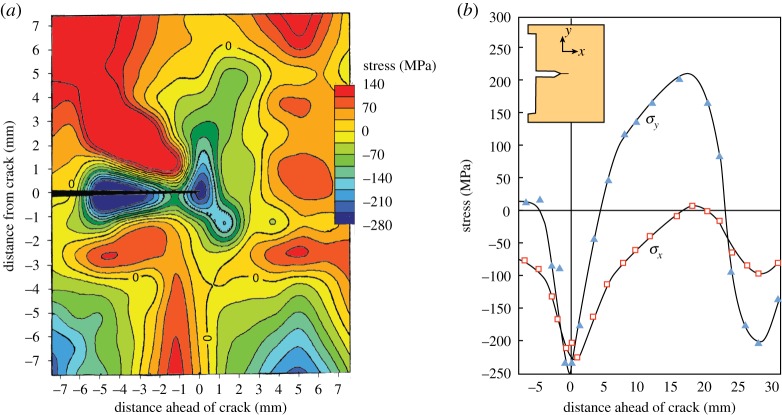
(*a*) A crack-opening residual stress map collected using laboratory X-rays with a 0.6 mm X-ray spot size for a 1020 steel after a 45 MPa√m overload during fatigue cycling at 16.9 MPa√ m showing very significant compressive stresses in the region of the crack tip. (After [15].) (*b*) Crack-tip residual stress field determined at mid-thickness of a 19 mm thick ferritic steel test piece (shown inset) by neutron diffraction using a ±3 mm cuboidal gauge, again showing the compressive residual stresses at the crack tip [16]. (Online version in colour.)

X-ray (figure 2*a*) and neutron (figure 2*b*) diffraction have been used to probe the state of stress in the vicinity of fatigue cracks since the 1970s and the 1980s, respectively. X-ray measurements can achieve submillilitre resolution but are confined to surface measurements, whereas neutron diffraction allows one to probe the stress state inside the bulk, but at millimetre resolution [17–19].

The recent emergence of high-brilliance, hard X-ray beamlines at third-generation synchrotron sources allows crack-tip strain fields to be mapped deep within test pieces [20,21]. Further, the very high intensity and the absence of significant beam divergence means that very small beams can be used to illuminate the sample. Indeed, sample gauge sizes as small as tens of micrometres in the lateral dimensions are feasible, although the gauge size is often somewhat longer along the beam direction.

**Figure 3.**
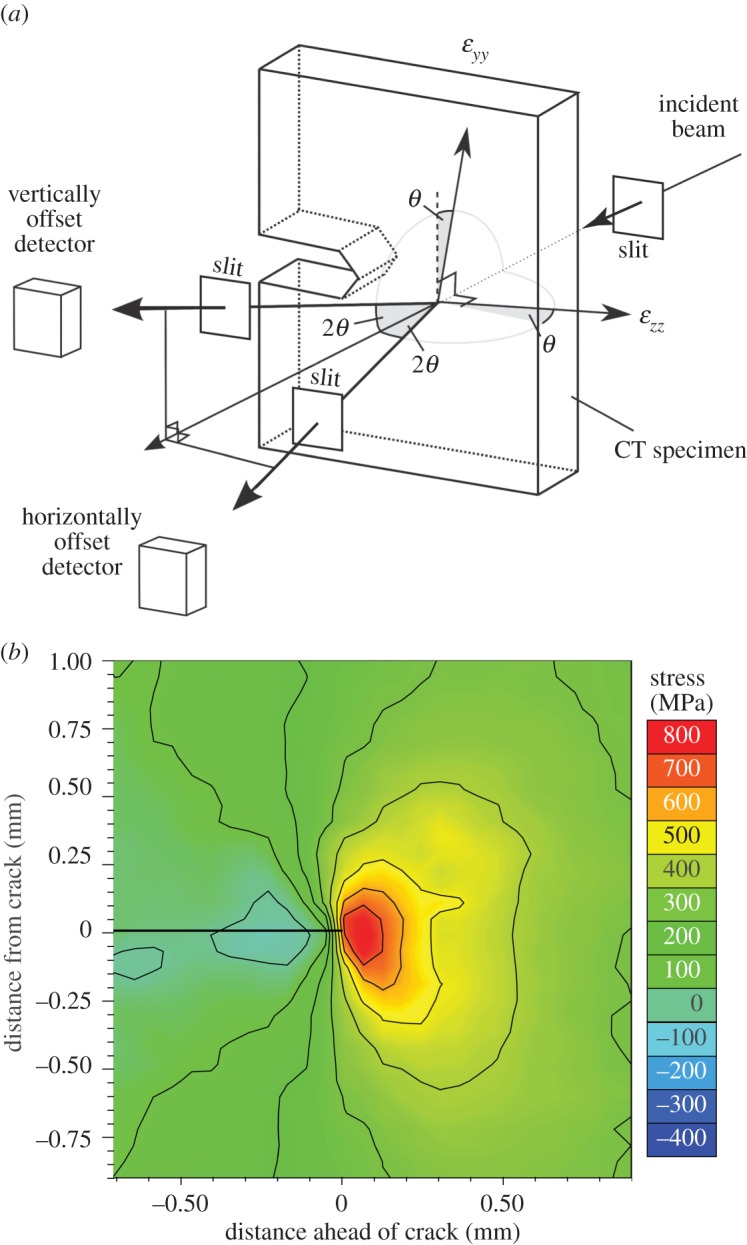
(*a*) Experimental set-up for collecting maps of the strains in the crack-opening (*ε*_*yy*_) and crack growth directions (*ε*_*zz*_) using energy dispersive detectors for a compact tension (CT) sample; (*b*) crack-tip stress field determined at the centre of a 12 mm thick CT test piece by synchrotron X-ray diffraction at *K*_I,max_ (13.2 MPa√ *m*) for an ultrafine grained (<1 μm) AA5091 Al alloy [22]. A 25×25 μm gauge cross-section was used. (Online version in colour.)

At the highest spatial resolutions, the need to illuminate a statistically significant number of polycrystalline grains for powder diffraction analysis means that the spatial resolution is in many instances limited essentially by the grain size of the sample rather than by the capability of the technique [12]. In such cases, this problem can be mitigated to some extent by oscillating the sample over a few degrees about the gauge volume location during each measurement so as to increase the number of diffracting grains that contribute. In the example shown in figure 3, the technique has been employed to study the crack-tip field in a thick compact tension sample having an ultrafine grain size (less than 1 μm) [22].

In this example, the stress field is essentially governed by LEFM, because the plastic zone size is very small, but there are many other more complex cases where the stress field local to the crack cannot be easily predicted, for example when there is extensive plasticity, crack deflection, a cohesive or bridging zone local to the crack, extensive microcracking or self-healing or when multiple phases are present. In such cases, elastic strain mapping is the most direct way of quantifying the stress experienced by the crack tip.

### (b) Mapping crack-tip stress-driven transformation effects

It has long been known that martensitic transformation effects can be used to improve the toughness of ceramics, such as partially stabilized zirconia (PSZ), by a mechanism called *transformation toughening* [23]. Here, the high stress concentration near a crack tip can trigger the transformation, in this case, of zirconia particles, introducing both a dilatation of around 4% and a shear strain locally. The typical transformation zone sizes found experimentally are of the order of 20 μm and the associated dilatation can cause the crack to close. The overall stress concentration near the crack tip is, therefore, reduced and thus the fracture toughness of the composite is enhanced [24]. This effect gives rise to an increasing resistance to fatigue crack growth with crack length (an increasing R curve response). Many studies have looked to characterize the process zone [25]. For example, it has been mapped by surface depression measurements by atomic force microscopy near the crack tip and by crack-opening displacement (COD) measurements. Indeed, COD measurements in PSZ [26] suggest that applied stresses as large as 400 MPa must be applied before there is significant crack opening. Despite these studies, to the author's knowledge, crack tip stresses in the transformed zone have yet to be mapped by diffraction.

**Figure 4.**
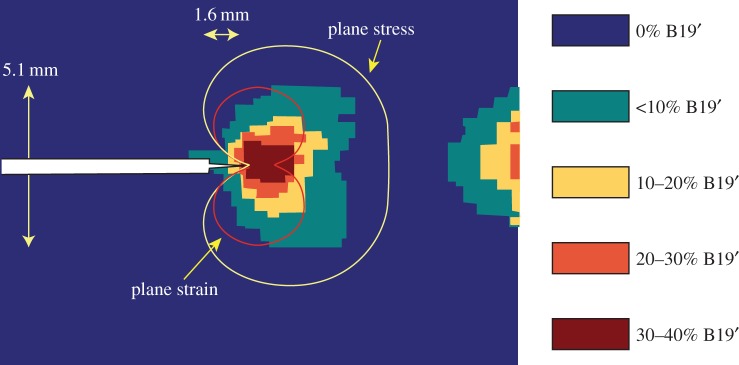
Phase fractions of austenite (B2) and martensite (B19' structures) near a crack tip in a pseudo-elastic NiTi shape memory alloy 8 mm thick CT specimen mapped by synchrotron X-ray diffraction [28] under a constant load. The austenitic plane stress and plane strain zones within which the transformation stress is exceeded are also shown. Note that material has also transformed near the back face owing to the large bending stresses there. (Online version in colour.)

The extent of the transformation has been quantified, however, for PSZ by X-ray diffraction on the fracture surfaces [27] and *in situ* at the crack tip for shape memory alloys [28] (figure 4). Shape memory materials have a similar capacity to transform locally under the crack-tip stress field. This shows rather elegantly the relationship between the local crack-tip stress field, the stress for transformation and the extent of transformation. To date, diffraction measurements have only scratched the surface of what can be achieved. Further, if combined with three-dimensional imaging (§4), a much more complete picture of the progress of transformation toughening with crack growth could be obtained providing crack-tip morphology, crack bridging and COD by imaging and the transformation zone and the extent of transformation and the crack-tip stress field by diffraction.

### (c) Plastic zone mapping

**Figure 5.**
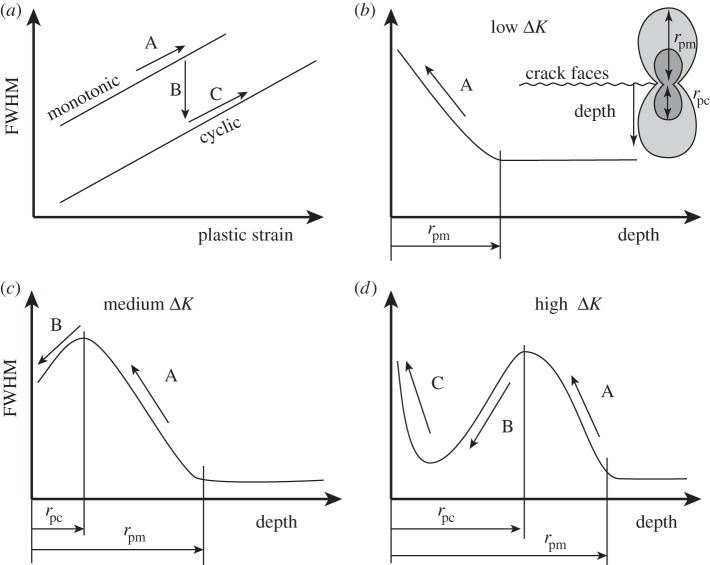
Schematic of the variation in the diffraction peak width, FWHM, measured by laboratory X-rays (*a*) with plastic straining and with depth from the fracture surface (*b*–*d*) with increasing levels of applied Δ*K* for a fatigue softening material. (After [32].) The relationships to the forward, *r*_pm_, and reverse, *r*_pc_, plastic zones are indicated.

**Figure 6.**
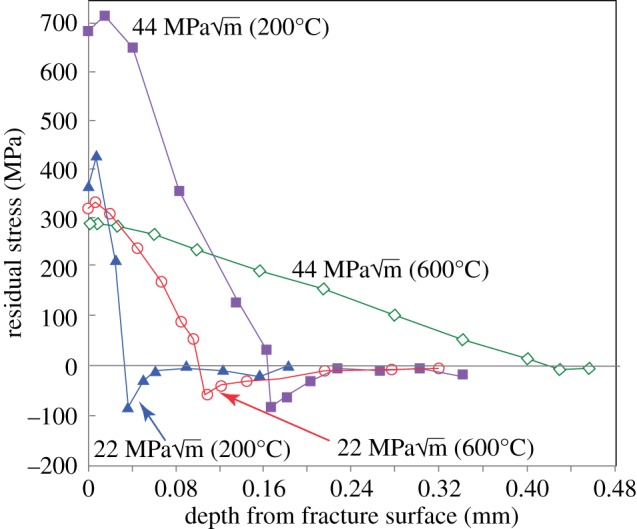
Depth profiles of residual stress measured in the crack growth direction by laboratory X-ray and successive layer removal over a 1×8 mm patch of the fatigue fracture surface by Cr *K*_α_ X-rays for AISI 4340 steel fatigued at two levels of *K*_max_ and two temperatures [29]. (Online version in colour.)

In the 1980s, the term *X-ray fractography* was developed to describe the use of X-ray diffraction to examine the fatigue fracture surface and the near-surface region by incremental electrochemical layer removal post-mortem [29–31] so as to obtain information on the crack-tip plastic zone. Two measures were proposed on the basis that the local plastic deformation at the crack tip leaves behind residual stresses and deformation on the fracture surface, namely the broadening of the diffraction line profiles and the residual stress parallel to the crack growth direction. The complex nature of the line broadening profile with depth is illustrated in figure 5 [32]. At low Δ*K*, the increasing peak width towards the crack surface reflects the increasing monotonic plastic strain (A). With increasing Δ*K*, the cyclic plastic strain increases such that fatigue softening (B) at the crack tip becomes important in the cyclic deformation zone, whereas at large Δ*K* the extensive cyclic cold work (C) at the crack tip may cause the full width at half maximum (FWHM) to rise. Similarly, complex behaviour has been observed for the variation in residual stress as a function of depth (figure 6). As a result, the profile shapes were not in themselves found to be a good indicator of the magnitude of the fatigue loading.

By contrast, the depths (*w*_*y*_) of the diffraction peak broadened region and especially the residually stressed zone were found to be good indicators of the extent of the forward plastic zone, *r*_pm_. Consequently, this could be related to the maximum applied stress intensity with [29–32]

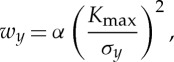
where *α* is a constant less than 0.19 [32] and *σ*_*y*_ is the yield strength.

Of course, the method can also be applied using synchrotron X-rays, but the increased penetration into the surface can complicate the depth analysis using a reflection geometry [33]. This approach can, however, easily be adapted to exploit the penetration of hard X-rays, by using a transmission arrangement (using a similar arrangement to that used for stress mapping in figure 3*a*), so that the different depths beneath the fracture surface (crack plane) can be probed non-destructively. Consequently, this technique need not be confined only to post-mortem fractography analysis but could be applied *in situ* during fatigue crack growth.

**Figure 7.**
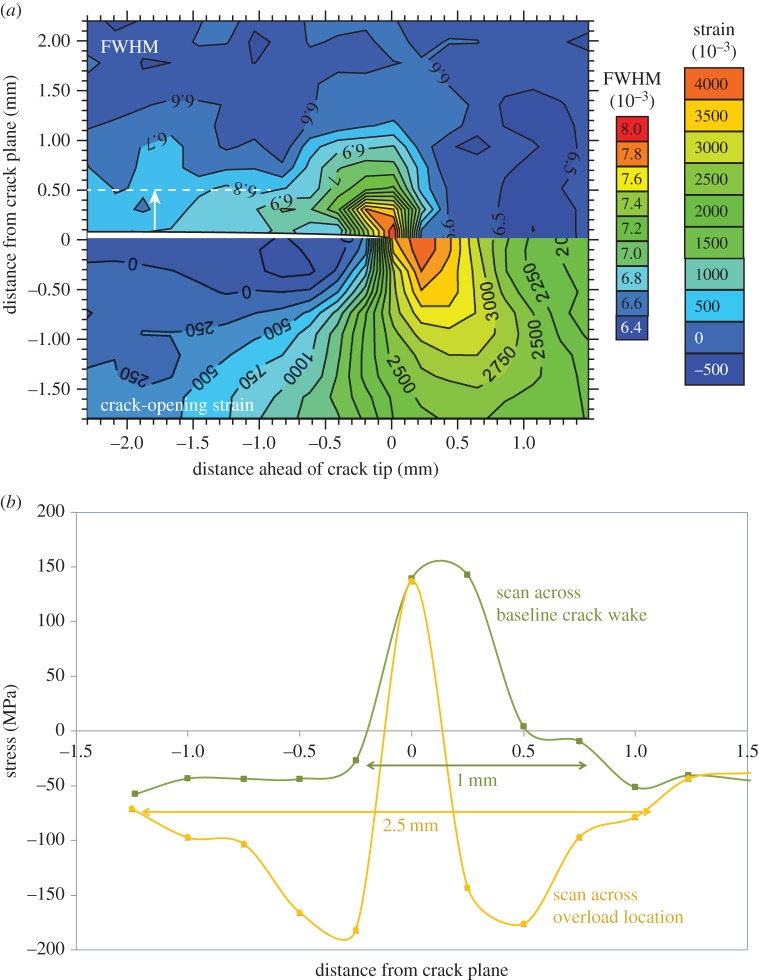
(*a*) Full width at half maximum variation (FWHM) (top) and crack-opening strain field (bottom) mapped local to a crack tip in a thick (15 mm) steel sample at *K*_min_ and *K*_max_(Δ*K*=28 MPa√m,*R*=0.05) using a 60×60 μm beam. Much of the broadening local to the crack tip is transient and progresses with the crack tip. (*b*) The variation in the residual stress parallel to the same crack plane at *K*_min_ across the crack faces for a region of the crack representative of growth at baseline fatigue and across the location of a 100% overload event. All data taken from experiments described in [34] on the energy dispersive beamline ID15 with FWHM expressed as Δ*E*/*E*_hkl_, where *E*_hkl_ is the X-ray energy at which the diffraction peak is centred. (Online version in colour.)

Data from the experiments looking at overloads during fatigue crack growth in bainitic steel CT samples described by Lopez-Crespo *et al*. [34] have been reanalysed and plotted. Figure 7*a* shows the variation of peak FWHM (top) alongside the elastic strain in the crack-opening direction (bottom) for a fatigue crack held under load at *K*_max_. It is clear that the peak FWHM variation is heavily influenced by the crack-tip strain field. The fact that the broadening is observed to vary significantly with loading condition and to follow the crack tip suggests that this broadening is not primarily owing to the dislocations associated with the crack-tip plastic zone (so-called type III microstresses). This broadening arises from the steep gradients in residual stress sampled across the 60 μm beam dimension. This is evidenced by the fact that the peak broadening is a maximum not at the position of maximum elastic (and plastic) strain (figure 7*a* (bottom)) but occurs just behind this where the gradient in strain is at its steepest. The diffraction peak width in the crack wake should be directly comparable to the conventional X-ray fractographic analysis. Here, the line profile is slightly broader than the far-field level extending to a depth (dashed line) of around approximately 0.5 mm from the crack faces (figure 7*a* (top)) which is probably indicative of the depth of the plastic zone.

By analogy with the laboratory X-ray method of figure 6, the variation in residual stress in the crack growth direction across the crack wake is shown in figure 7*b*. This is also broadly consistent with a plastic zone of around approximately 0.5 mm for baseline fatigue. In the region of the 100% overload event, this has extended to approximately 1.25 mm. Using Irwin's equation [35] for the plastic zone (yield stress 570 MPa) would give values of 0.25 and 1 mm for the baseline and overload fatigue cases, respectively [36]. This suggests that the plastic zone depth can be used as an indicator of the crack driving force.

For materials for which there is a strain induced transformation local to the crack-tip, the extent of the plastic zone can also be determined by monitoring the relevant phase fractions. For example, the loss of austenite owing to the formation of deformation-induced martensite in the plastic zone has been used to assess the extent of the plastic zone for austenitic and duplex stainless steels [37,38]. Such transformations are also likely to further complicate the peak broadening and residual stresses profiles with distance from the fracture surface.

### (d) Crack-face tractions

A great deal of attention has been focused on the role of crack-face closure in fatigue crack-tip shielding. Despite the identification of closure as a potential crack-tip shielding mechanism by Elber back at the start of the 1970s [39], it is still the focus of much debate. Vasudevan *et al.* [40,41] suggest that many fatigue crack growth rate retardation effects can be explained in terms of changes in the stresses ahead of the crack tip rather than in the crack closure (such as plasticity, roughness or oxide-induced closure [42,43]) behind it. In particular, there is much debate regarding the possibility for closure in thick samples under plane strain [44,45]. Nevertheless, changes in crack compliance have been observed in many cases for plane stress samples [46–48] and these can be simply accounted for by the concept of closure.

#### (i) Closure stresses

**Figure 8.**
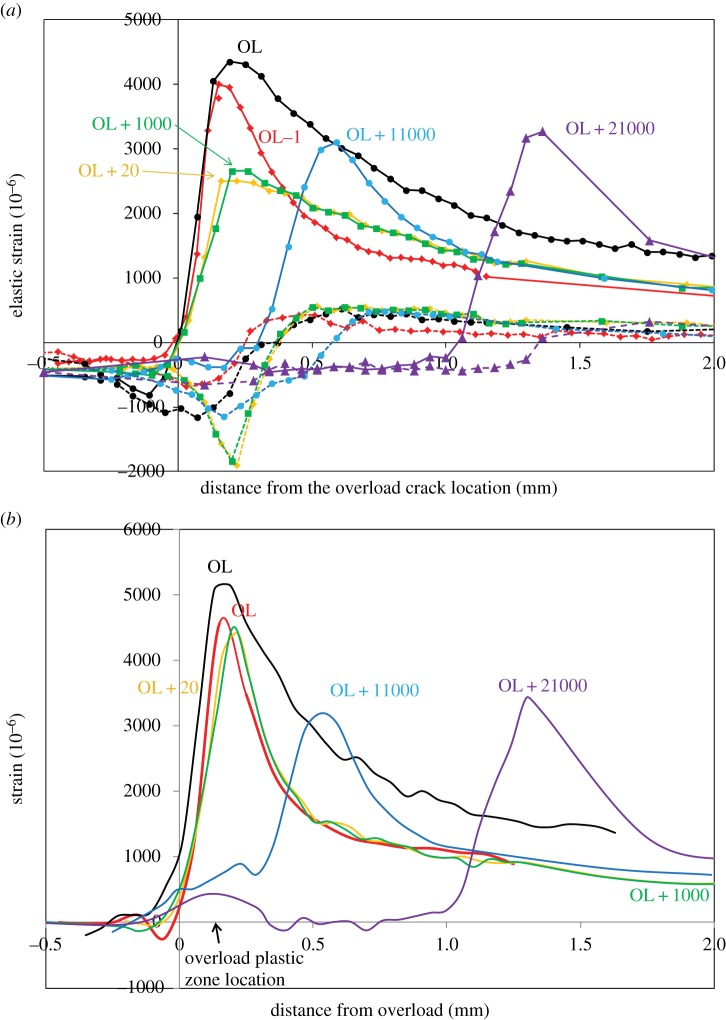
(*a*) Crack-opening strain, *ε*_*yy*_, profile along the crack plane (*y*=0) at the centre of a 15 mm thick bainitic steel CT sample at *K*_max_ (solid line) and *K*_min_ (dashed line; Δ*K*=28 MPa√m; R ratio=0.05) as a function of the number of cycles after a 100% overload (OL) event. (*b*) The same data plotted as the difference in elastic strain between *K*_max_ and *K*_min_ as a function of the number of cycles. The arrow shows the location of the extensive plastic deformation from the overload event [36]. (Online version in colour.)

In principle, diffraction provides a means of quantifying crack-face closure contact stresses. An example is provided by Lopez-Crespo *et al.* [36], who measured a series of line scans along the crack plane to follow the progress of the crack-tip elastic strain field (crack-opening strain) with number of cycles before, and after, a 100% overload (figure 8).

**Figure 9.**
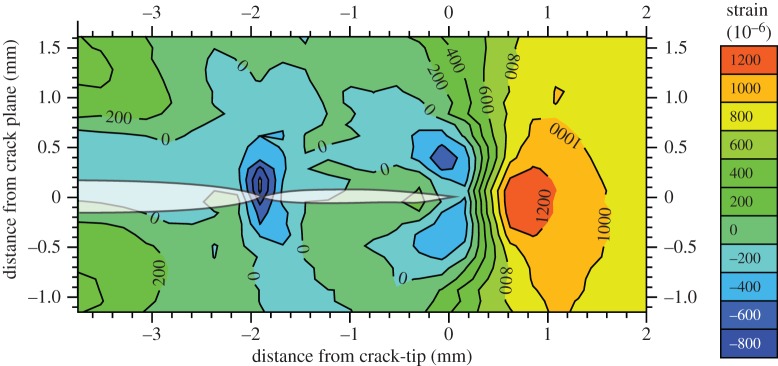
Two-dimensional map of the elastic strain (in 10^−6^) in the loading direction at *K*_min_(Δ*K*=28 *MPa*√m, R=0.05) showing the discontinuous contact strain field at the location of a 100% overload event (−1.9 mm behind the current crack position). (Courtesy of Crespo Lopez, Steuwer, Kelleher and Withers.) (Online version in colour.)

As one might expect, the overload event generates a large plastic zone. This causes the peak stress at *K*_max_ on reloading (OL+20 cycles) to be smaller than that prior to overload (OL−1 cycle; figure 8*a*). With further crack growth, the peak stresses rise as the elastic crack field grows out of the compressive plastic zone. Figure 8*b* illustrates that immediately after the overload event the elastic strain changes at the crack tip on loading from *K*_min_ to *K*_max_ are very similar to those immediately prior to overload. This indicates that the lower peak tensile stress after overload is primarily the result of the larger compressive stress in the plastic zone at the crack tip. Immediately after overload, it is difficult to discern any compressive stress being transferred across the crack faces; however, it is evident that significant compressive residual stresses do act across the crack faces at the location of the overload event when the crack has grown some distance past it (figure 8*b*). This effect has been proposed previously and referred to as discontinuous closure [45] whereby plastic ‘wedges’ are left on either side of the crack flanks which come into contact during unloading as the crack grows through the initial extended forward plastic zone. It is more clearly seen in two-dimensional maps of the stress field at *K*_min_ as shown by [34] for a similar experiment in figure 9.

#### (ii) Bridging stresses

**Figure 10.**
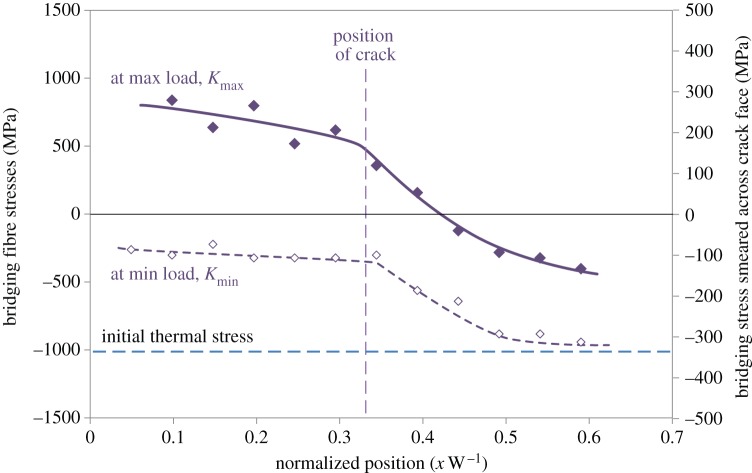
Fibre bridging stresses for the SiC fibres bridging a fatigue crack in a Ti–6Al–4V/35% SiC long-fibre composite holding the crack shut at *K*_max_ (filled symbols, continuous lines) and propping it open at *K*_min_ (open symbols, dashed curves). The far-field thermal contraction mismatch residual stresses in the fibres are represented by the horizontal dashed line. (After [50].) (Online version in colour.)

While closure stresses act to keep cracks open at *K*_min_, ligaments that bridge the crack can help to hold a crack shut at *K*_max_ thereby reducing the cyclic amplitude (Δ*K*^eff^) experienced by the crack tip. Such crack-face tractions can also be evaluated by diffraction. This is well illustrated by the role of bridging fibres in shielding a fatigue crack in long-fibre composites. The bridging stresses have been quantified by high-resolution synchrotron diffraction for Ti–6Al–4V/35% SCS-6 SiC fibre composite [49]. The bridging stresses in the fibres are shown in figure 10. The crack-face tractions arising from the bridging fibres are around 300 MPa at maximum load. Interestingly, they are compressive (approx. −100 MPa) at *K*_min_ which tends to prop open the crack. This is partly due to the fact that the bridging fibres are pulled out somewhat at *K*_max_ and partly due to the original compressive thermal residual stresses in the fibres. Using a weight function approach, it is possible to calculate the shielding effect of the crack-closing stresses at *K*_max_ and the crack-opening stresses at *K*_min_. These are shown in §5*b* and act to greatly reduce the stress intensity range experienced by the crack as more and more fibres bridge the growing crack.

## 3. Crack-tip imaging

As discussed by Stock [51], the narrowest crack opening that can be measured by tomography is a function of the pixel size and the contrast difference. For aluminium viewed with a 40 kV laboratory source, Breunig *et al.* [52] suggest that a crack as small as 10% of the pixel size can be measured. For polymer composites illuminated with X-rays from a 100 kV source, a sensitivity to cracks 20% of the pixel size has been quoted [53] improving to 5% if dye penetrant is used to enhance the contrast. However, such values appear rather optimistic under all but the most favourable conditions. Further, crack detection can be assisted by imaging under load such that the cracks are held open. In any event, provided the cracks can be detected, X-ray tomography can provide a feast of information as to the mechanisms of damage occurring ahead of the crack and crack-tip shielding behind it as illustrated in figure 1 and discussed below.

### (a) Time-lapse CT

**Figure 11.**
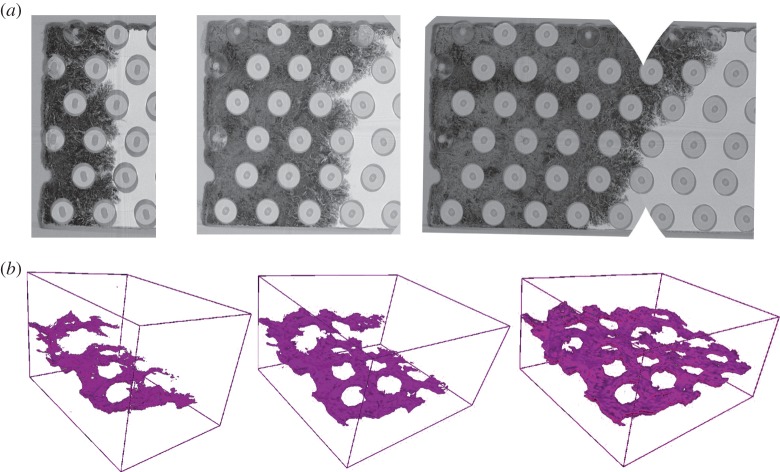
(*a*) Plan view and (*b*) three-dimensional view of the progressive growth of a fatigue crack in a Ti matrix-C cored 140 μm SiC fibre composite with the matrix and fibres rendered transparent in both views. (After [50].) (Online version in colour.)

One of the advantages of tomographic imaging is that it is non-destructive and so can be applied repeatedly over time to follow the progress of a crack (figure 11). This is sometimes called four-dimensional imaging, or, more straightforwardly, time-lapse CT. A good example of the power of the method is provided by the progressive damage introduced in a [90/0]s carbon fibre-reinforced laminate as a function of straining [54]. Considerable work has also been done studying the progress of fatigue cracks [50,55–63]. The method is not just confined to the study of laboratory test pieces; in a number of cases crack propagation has been followed in components or devices. For example, Tsuritani *et al.* [64] have studied the development of cracks in 150 μm solder microbumps of a flip–chip interconnect. In this case, refraction (phase) contrast [65] has been used to increase the contrast, because the cracks open by less than 100 nm. In this way, it was possible to monitor the growth of cracks between the solder and the Cu pad over repeated thermal cycles to failure.

### (b) Short cracks

It is well known that microstructurally short cracks are very sensitive to the local microstructure. In regions where the local microstructure is favourable for growth, much faster growth rates can be achieved than would otherwise be expected. Conversely, Buffiere *et al.* [66] found by tomography that at low stress levels fatigue crack growth from pores could be held up by microstructural barriers such as grain boundaries, whereas at high stress levels continuum crack growth laws were well obeyed. Other studies have looked at short crack/grain boundary interactions. Because grain boundaries are not normally visible using conventional attenuation tomography, grain boundary decoration techniques have been applied (e.g. aluminium grain boundaries decorated using Ga) [67–69]. These were accompanied by post-mortem electron back-scattered diffraction analysis so as to be able to relate the grain structure and grain orientations to the crack path under fatigue.

**Figure 12.**
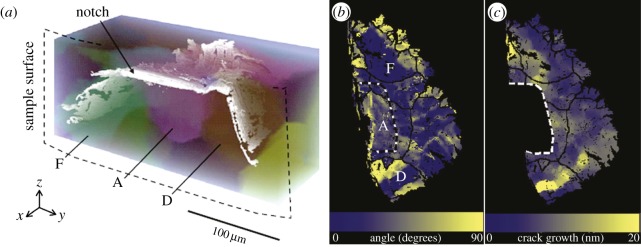
(*a*) Three-dimensional diffraction contrast tomography rendering of a crack (white) and Mg grains (semi-transparent colours); (*b*) plan view of the same crack with the starter notch (dashed), grain boundaries (lines) and specific grains labelled by letters showing the angle between the crack plane and the crystallographic basal plane; (*c*) local growth rate (nm per cycle) where the average rate was 9 nm per cycle [72]. (Online version in colour.)

More elegantly, a combination of diffraction and tomography can provide information about the grains and their orientations non-destructively alongside attenuation CT imaging of the crack. For example, diffraction contrast tomography [70] or three-dimensional X-ray diffraction [71] are being used to map the grain orientations in three dimensions. A good example is the study of stage I crack growth in magnesium (figure 12). This was found to occur preferentially on the basal plane of the hexagonal close-packed crystal, whereas retardations in the local crack growth rate were observed at boundaries with large misorientations to the slip plane [72].

## 4. Monitoring extrinsic toughening mechanisms

The intrinsic toughness of a material relates to its inherent ability to absorb energy as a crack grows, for example by plasticity. Extrinsic toughening can be introduced by the addition of second phases, heterogeneity or controlled interfaces into the microstructure. There are a large number of extrinsic toughening mechanisms which can shield the crack tip. These have been categorized as crack deflection, those which arise in a zone local to the crack tip referred to as ‘zone shielding’, and closure mechanisms [73] that tend to act across the crack wake. The extent to which imaging can provide information about the activation of each of these mechanisms is considered, in turn, in the following sections, although for many heterogeneous systems multiple toughening mechanisms operate simultaneously.

### (a) Crack deflection and meandering

The effect of texture in terms of crack deflection by microcracking has been investigated by tomography for some time including Al–Li alloys [74] and iron titanate ceramics [75]. The work of Stock is particularly noteworthy as he has combined X-ray tomography and diffraction, although on different instruments, using microbeam diffraction to quantify the role of grain orientation on grain deflection identified by imaging and finding that specific orientations of similarly oriented grain clusters produce sharp changes in crack path.

Many natural materials are extremely anisotropic, giving rise to extensive crack deflection, for example wood, seashells, bone [76], bovine enamel/dentin [77] and elephant tusk [78]. For example, for bone (see §4*c*(i)) three-dimensional imaging of cracks growing transverse to the length of the bone shows marked crack deflections and (out-of-plane) twists as they interact with the underlying Haversian structure [79,80]. This is an important source of toughening for cracks grown in this orientation.

**Figure 13.**
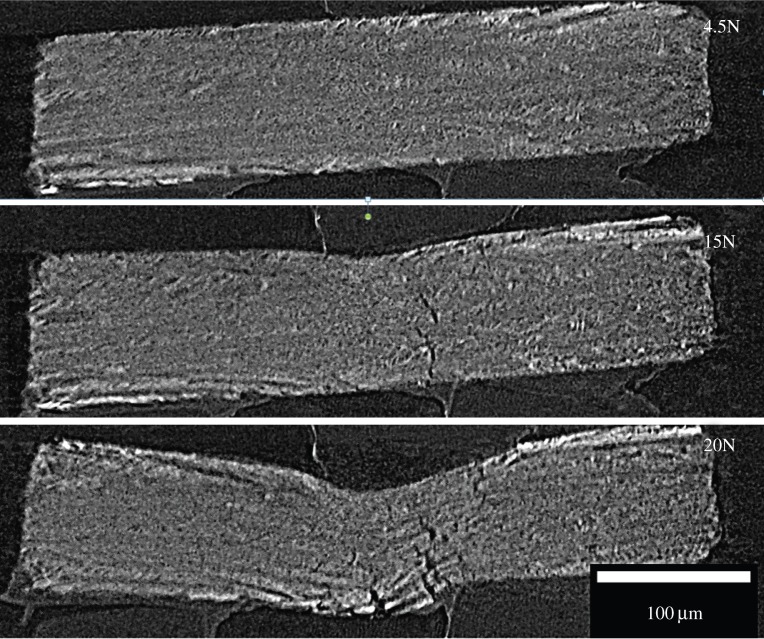
Time-lapse synchrotron X-ray tomographic cross-sections (I13 Diamond Light Source) showing crack deflection in a freeze cast layered ceramic (approx. 4×0.8×1.6 mm) fractured in bending. (Courtesy of D Eastwood, E. D'Elia, E. Saiz. S Yue, C. Rau, P.J. Withers and P. D. Lee.)

Recently, there has been a great deal of interest in mimicking the layered structured of many natural systems, originally on the submillimetre scale by stacking thin ceramic sheets [81] and more recently at the tens of micrometres scale by freeze casting (ice templating) [3,82]. Time-lapse CT is especially well suited to the characterization of the crack deflection events and can provide key information about the sequence of crack growth, bifurcation and deflection as well as the opening of the dominant crack, as shown in figure 13.

### (b) Crack-tip zone shielding

**Figure 14.**
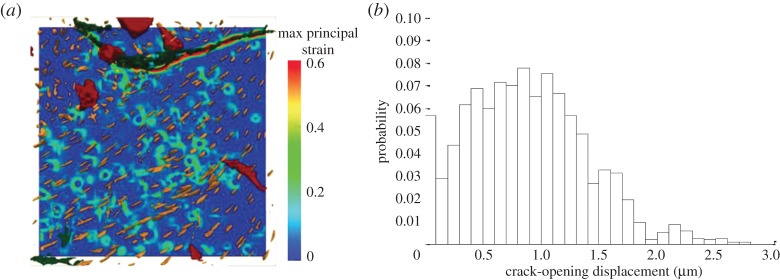
(*a*) The strain map in the transverse plane for a mouse femur just before failure with the osteocyte lacunae (yellow) aligned out of plane and homogeneously spaced approximately 20 μm apart in which the microcrack (green) is propagating from top right to left. The out-of-plane canal (red) causes a stress concentration and, consequently, an upwards deflection of the microcrack. (*b*) The histogram of the crack-opening displacement (COD) just before failure pooled from many samples. The opening of the microcracks reaches a plateau (95% limit at 1.74 *μm*), where further energy is dissipated only by other mechanisms, such as the extension of the crack or by other cracks [87]. (Online version in colour.)

A number of mechanisms have been proposed by which crack-tip zone shielding can occur, including transformation toughening (see §3*b*), microcrack toughening, crack-wake plasticity, void formation and residual stress [73]. In many cases, the efficacy of these in terms of lowering the crack-tip stress intensity can now be assessed quantitatively by diffraction through their effect on the local stress field (§3*a*). Relatively little work has been done examining microcracking by tomography, though it has been reported for bone [83,84] and in tusk dentin [85]. Voide *et al.* [86] and Christen *et al.* [87] have studied microcracking in bone by time-lapse CT. They found that microcracks often initiated at stress-concentrating canals (figure 14*a*) and that osteocyte lacunae were able to guide microcrack propagation affecting their orientation and direction. Using digital volume correlation Christen *et al.* found that microcracks were only visible at far higher tensile strains (more than 15%) than that assumed to initiate plastic deformations, suggesting a considerable ability of bone to accumulate damage at the tissue level prior to the initiation of microcracks. They also found that the COD increased only up to approximately 2.0 μm with increased loading (figure 14*b*). Instead of further crack opening, the energy is then released by either an increase of the crack length or the formation of additional microcracks. In many cases, such cracking ahead of the crack tip can also lead to crack bridging, which is often a more significant shielding effect, as indicated by previous studies [76,88].

### (c) Contact shielding by crack bridging

**Figure 15.**
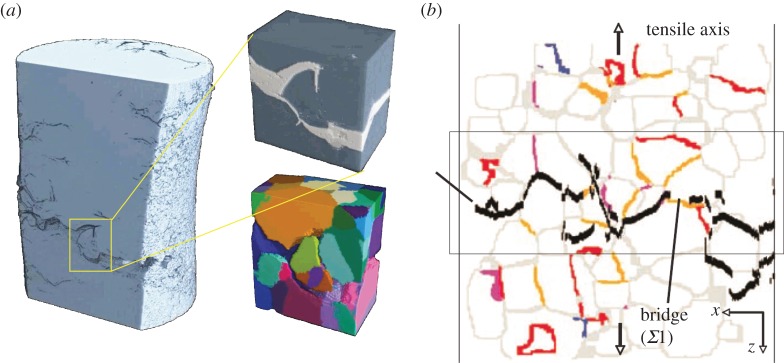
Combined use of diffraction contrast tomography (DCT) and CT data to identify crack-bridging grain boundary structure for intergrannular corrosion in a stainless steel wire. (*a*) Integranular cracking is evident from the segmented attenuation contrast image (top) but the relationship to the underlying grain structure is only evident from the magnified DCT image (bottom). (*b*) Two-dimensional section of the grain boundaries identified by DCT compared with the path identified by CT. The low-angle boundaries *Σ*1 (orange), *Σ*3 (red), *Σ*9 (blue) and other boundaries <*Σ*29 (purple) [70]. (Online version in colour.)

Interfaces or discontinuities, arising between distinct phases, from material anisotropy, or due to porosity or defects, can lead to uncracked bridging ligaments that shield the crack front from the applied load. In some cases, this is by the creation of smaller microcracks ahead of the crack that link with the main crack or by forming crack-resistant islands (figure 15) after the main crack has passed. Crack bridging is one of the most important crack-tip toughening mechanisms. Because bridging arises across the crack wake, bridging mechanisms tend to cause the crack resistance to increase with crack length (R-curve behaviour).

#### (i) Crack bridging

*Grain-scale bridging.* Crack bridging is important not just for fatigue cracking, but also in understanding intergranular stress corrosion cracking. Here, the properties of the individual grain boundaries are paramount in determining the extent of crack bridging that is achieved. Diffraction contrast tomography enables the grain orientations to be measured, and the nature of the individual grain boundaries to be inferred and related to their susceptibility to cracking (figure 15).

#### (ii) Interface-controlled bridging

**Figure 16.**
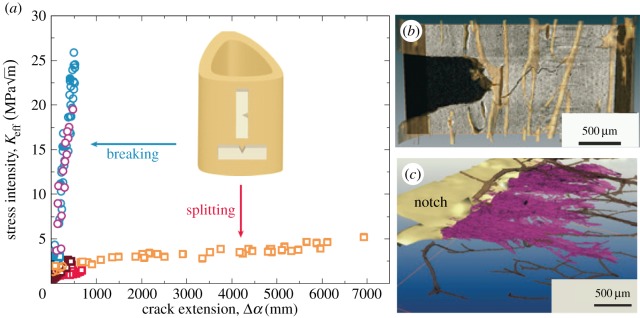
(*a*) Fracture toughness resistance curve data for the transverse and longitudinal orientations in hydrated human cortical bone [89] and tomographs showing the crack growth path for cracks growing (*b*) transverse to the axis of the bone [80] and (*c*) along the length of the bone [89]. In (*b*) crack deflection/twisting dominates and in (*c*) crack bridging dominates the toughening. (Online version in colour.)

Interfaces can hold up crack growth or initiate new cracks to which the main crack will link up through the formation of bridges. The latter can be achieved by controlling the interface properties or by the exploitation of defect structures. These effects are used widely in nature; for example, for cracks growing along the length of bone (figure 16) microcracks tend to form ahead of the crack along the lines of the Haversian canals which then give rise to crack bridging as they join the primary crack. The increased osteonal density in older bone leads to smaller and less frequent crack bridges and correlates with the marked reduction in the slope of the R curves with age [76]. This mechanism is less effective than the crack deflection that arises when a crack grows perpendicular to the length of the bone. In contrast to bone, dentin has been observed to be toughest for cracks grown parallel to the tubules; this suggests that the significant effect on toughening is not from the bridging of the tubule lumens, but rather from the bridging between microcracks initiated from the tubules [90].

Perhaps the most significant form of toughening by crack bridging arises when ceramic fibres reinforce metallic matrices where the interface strength is sufficiently low to allow crack deflection along the fibre matrix interfaces. A good example is provided by aligned Ti/SiC fibre composites where very significant crack-bridging stresses arise, as discussed in §2*d*.

#### (iii) Observing crack-face contact

As discussed in §2*d*, a key aspect in terms of understanding macroscopic fatigue crack growth is crack closure [39]. Stock and co-workers [91–94] were among the first to quantify the extent of crack-face contact (closure) during fatigue loading of aluminium alloys. From this work, they concluded that, as one might anticipate, planar sections of cracks tend to close relatively uniformly, whereas asperity-dominated surfaces tend to close at irregular locations. More importantly, they found that considerable physical crack closure during unloading precedes the knee in the load–deflection curve traditionally ascribed to crack-face closure. Consequently, asperities can give rise to closure at stresses surprisingly near to *K*_max_ (see §5*d*). A number of researchers have subsequently used X-ray tomography to evaluate closure effects [57,68,95,96]. The mechanical consequence of closure is probably more affected by the subsequent onset of significant load carrying across the surfaces (figure 9) than the moment of first contact.

## 5. Quantification of the crack-tip driving force

While maps of the stress field and images of the predominant damage mechanisms allow materials designers to infer much about the efficacy of various toughening mechanisms, quantitative measurements of the crack driving force provide useful measures of the efficacy of the active toughness mechanisms. Historically, a number of parameters have been used to quantify the crack driving force, including the elastic energy release rate, *G*, the stress intensity factor, *K*, the *J* integral, the CTOD and the crack-tip opening angle (CTOA). All have their merits [97]: generally *G* and *K* are suited to elastic or nearly linearly elastic materials, whereas CTOD and *J* integral are better suited to elastic–plastic materials. All of these except for *G* can be evaluated by synchrotron diffraction, imaging, or both.

### (a) Crack driving force inferred from the elastic strain field

**Figure 17.**
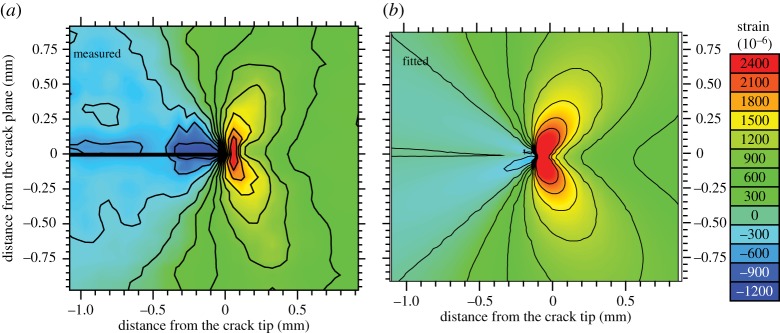
The measured (*a*) and best-fit (*b*) elastic strain fields (in 10^−6^) for the crack-opening (*ε*_*yy*_) direction at *K*_Imax_=6.6 MPa√m for an Al 5091 CT sample. The LEFM best-fit crack-tip strains correspond to a crack-tip stress intensity of *K*^eff^_Imax_=6.11 MPa√m,K^eff^_*IImax*_=0.33MPa√m (table 1) [22]. (Online version in colour.)

By comparing the measured crack-tip strain field directly with that predicted by LEFM, it is possible to infer the stress intensity factor effective at the crack tip, *K*^eff^, directly from the local elastic strain field [22], as summarized in table 1 for the strain field in figure 17. It is evident from the table that in this case, where the plastic zone is small, the nominal stress intensity calculated from the crack length, the applied load and the specimen geometry is in very good agreement with that actually experienced by the crack tip. One of the disadvantages of fitting to the *K* field is the need to accurately know the location of the crack tip [98].

**Table 1.** Stress intensity factors (in units of *MPa*√m) as nominally applied from the far-field load and inferred at the crack tip by fitting LEFM solutions to the strain maps measured mid-thickness at maximum load during a normal cycle and during an overload cycle for an AA 5091 sample [22]. The uncertainties given in the parentheses are 2×standard deviation (95%) confidence limits.

fatigue/overload	applied *K*^nom^_I_	crack tip *K*^eff^_I_	crack tip *K*^eff^_II_
fatigued	6.6	6.11 (0.21)	0.33 (0.53)
100% overload	13.2	13.3 (1.83)	0.71 (0.38)
one cycle after overload	6.6	6.2 (0.46)	−0.06 (1.19)

### (b) Crack driving force inferred from the crack-face tractions

**Figure 18.**
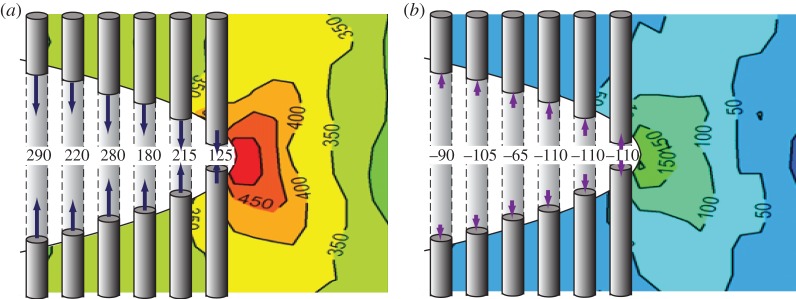
The fibre stresses measured at the fatigue crack plane shown in figure 10 for a Ti–Al–4V/35% SiC fibre composite can be considered as crack-face tractions at (*a*) *K*_max_ and (*b*) *K*_min_. These crack-closing (*a*) and crack-opening (*b*) stresses are shown superimposed upon the measured continuum crack-tip stress field (in MPa; after [50]) derived by summing the stresses in each phase in their correct phase fractions. The crack length is approximately 0.35 through the width of the sample. (Online version in colour.)

**Figure 19.**
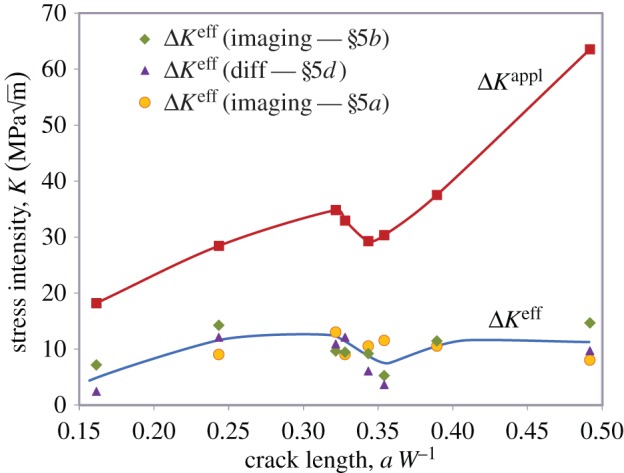
Increase in crack-tip driving force with crack length compared with that nominally applied (red squares) for a 35vol% SiC fibre/Ti alloy composite as inferred from the crack-tip stress field (orange circles) using the approach of §5a, from the stresses in the bridging fibres (green diamonds) as described in §5b and the local crack-opening displacements (purple triangles) as described in §5d. (After [49].) (Online version in colour.)

Often, measurements of the COD are used to infer the crack-face tractions [99]. However, in some cases, the crack-face tractions can be determined and the effective stress intensity range can be calculated either using weight functions [100] or by finite-element methods [101]. In some cases, diffraction can provide a direct measure of the bridging stresses. By way of an example, consider the fibre stresses shown in figure 10 for a Ti–Al–4V/35% SiC fibre composite. The net effect of these fibres can be considered as crack-face tractions acting over the crack faces, as shown in figure 18. These crack-face tractions can then be used to infer the stress intensity range effective at the crack tip, as shown in figure 19. What is clear from the figure is that as the crack grows more and more fibres bridge the crack, so that, ultimately, the fibres either break or arrest the crack [10,50].

### (c) Crack driving force inferred from the displacement field

**Figure 20.**
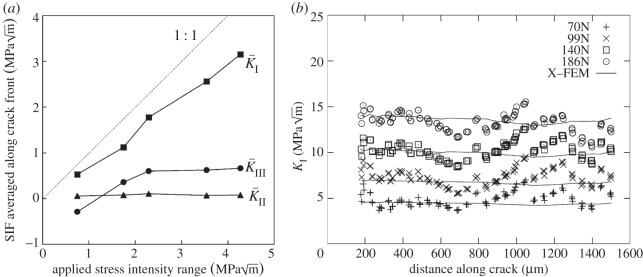
(*a*) Variations of modes I, II and III local crack driving forces as a function of applied stress intensity range determined from crack-opening displacements measured by tomography. The data were averaged along the whole crack front of an Al alloy single-edged notch sample. The theoretically applied SIF was pure mode I and given by the straight line [102]. (*b*) Extended finite-element modelling (X-FEM) and mode I stress intensity factor along the crack front after 45 000 cycles as deduced by digital volume correlation based on the nodules in nodular cast iron [103].

Toda *et al.* [102] were probably the first to infer the crack driving force directly from the point-to-point displacements during the opening and closing of a crack measured in three dimensions from the movement of markers (often inclusions or micropores) near the crack tip (figure 20a). This is normally expressed in terms of the relationship between displacement and the stress intensity factor *K* for small-scale plasticity or the *J*-integral for larger-scale yielding. This approach has been taken further through the use of digital volume correlation to map three-dimensional displacements; the variation in the local crack-tip driving force can be inferred all along the crack front by three-dimensional modelling [95,103] (figure 20b).

### (d) Crack driving force inferred from the crack-tip opening displacement

The change in CTOD during a fatigue cycle is an important measure of the crack driving force. One of the first teams to measure the CTOD as a function of the load was led by Stock [91], identifying asperity contact well before the knee in the traditional compliance curve. Subsequent higher-resolution measurements by Toda *et al.* [59] found a linear relation between the CTOD and the applied stress intensity with crack-face contact arising largely from a small mode III displacement, reducing continuously upon reloading with small regions of contact even at *K*_max_. The role of crack bridging by unbroken bridging fibres on the COD variation in a metal matrix composite has also been mapped [55].

Withers *et al.* [49] have recently brought together imaging and diffraction measurements of crack-tip driving force for fatigue crack in a Ti/35 vol% SiC fibre composite in a key demonstration of the complementarity of diffraction (crack-tip stress field and fibre-bridging stresses) and tomography (COD measurement) to infer the crack-tip driving force throughout fatigue cycles (figure 18). Note from the figure how the actual stress intensity experienced by the crack tip stays approximately constant as the crack grows despite the sharp increase in the applied nominal stress intensity as the cracked section increases.

## 6. Conclusion

To conclude, just as the imaging and diffraction modes of a transmission electron microscope are used in tandem to interrogate materials microstructures, the synchrotron X-ray diffraction and computed tomographic imaging modes offer the prospect of delivering complementary information across a range of engineering materials problems. This review has explored the extent to which diffraction can provide information about the stress field local to the crack as well as the extent of local plasticity and transformation, whereas imaging can provide a great deal of information about the activation of crack-shielding mechanisms, crack deflection and propagation or even crack healing. In many cases, multiple toughening mechanisms operate, and it can be difficult to infer the effectiveness of each simply by imaging their activation. The examples given in this paper show how, taken together, diffraction and imaging enable a number of key fracture mechanics parameters such as the stress intensity factor, *K*, the *J* integral, the CTOD and the CTOA to be quantified. This information promises to improve our understanding of crack growth, leading to better structural integrity assessments, as well as guide materials designers to exploit crack-toughening micromechanisms.

To date, examples where X-ray imaging and diffraction have been combined are rare owing to the lack of suitable instruments. One example is provided by the study of the pores that develop during the heat treatment processing required to produce superconducting Nb_3_Sn strands in copper [104]. By comparing the evolution of void volume (observed *in situ* by imaging) with that of the various phase fractions (measured by diffraction), it was shown that the pores are formed because of density changes upon the formation of certain intermetallic phases.

Looking to the future, there are many cases where a combination of diffraction and imaging could provide complementary insights in three dimensions, particularly where changes in stress or the occurrence of phase transformations need to be related to structural changes or degradation. Mixed methods such as diffraction contrast tomography will enable both three-dimensional crystallographic information and strain at the grain-by-grain level to be co-visualized with three-dimensional damage or structural information from imaging. It will be possible to use fast tomography to survey regions to identify defects or features of interest, around which detailed stress mapping may be useful in either two or three dimensions.

The highly penetrating nature of synchrotron X-rays will enable the interaction between local stress concentrations and the onset of failure to be explored *in situ* over time under demanding environments typical of particular in-service conditions, for example

— to understand the load redistribution (by diffraction) that takes place locally in long-fibre composites under static or fatigue loading when one or more fibres break (as recorded by imaging) [105];
— to quantify the relationship between stress relaxation [106] and creep cavitation [107];
— to study the accumulation of damage (by imaging [108]) in piezoelectric actuators/ferroelectric devices as a function of phase transformations and residual stress (quantified by diffraction [109]) under alternating electric field and/or load;
— to follow fatigue crack growth (imaging) in shape memory materials (recording the phase transformations and stress by diffraction) [110,111];
— to follow the local oxidation (by diffraction) and crack-healing kinetics (by imaging) during crack self-healing at elevated temperature for a MAX phase ceramic [112]. Subsequently, the ability of the healed crack to bear load prior to cracking could also be assessed by diffraction.

Almost certainly, there are many more, as yet unforeseen, applications of diffraction and imaging. More beamlines such as ID15 at the ESRF and JEEP at the Diamond Light Source need to be developed that allow both techniques to be applied sequentially or simultaneously on the same sample. Further, the ability to acquire such information quickly [113], to extract quantitative information [114] from the images and to correlate information across scales with complementary three-dimensional information from other instruments [115] will allow a much wider range of *in situ* experiments to come under the microscope.

## References

[1] Ritchie RO 1999 Mechanisms of fatigue-crack propagation in ductile and brittle solids. Int. J. Fract. 100, 55–83. (10.1023/A:1018655917051)

[2] Launey ME Munch E Alsem DH Barth HB Saiz E Tomsia AP Ritchie RO 2009 Designing highly toughened hybrid composites through nature-inspired hierarchical complexity. Acta Mater. 57, 2919–2932. (10.1016/j.actamat.2009.03.003)

[3] Launey ME Munch E Alsem DH Saiz E Tomsia AP Ritchie RO 2010 A novel biomimetic approach to the design of high-performance ceramic-metal composites. J. R. Soc. Interface 7, 741–753. (10.1098/rsif.2009.0331) 19828498 PMC2874234

[4] White SR Sottos NR Geubelle PH Moore JS Kessler MR Sriram SR Brown EN Viswanathan S 2001 Autonomic healing of polymer composites. Nature 409, 794–797. (10.1038/35057232) 11236987

[5] Stefanescu D Steuwer A Owen RA Nadri B Edwards L Fitzpatrick ME Withers PJ 2004 Elastic strains around cracked cold expanded fastener holes measured using the synchrotron X-ray diffraction technique. J. Strain Anal. 39, 459–469. (10.1243/0309324041896470)

[6] King A Steuwer A Woodward C Withers PJ 2006 Effects of fatigue and fretting on residual stresses introduced by laser shock peening. Mater. Sci. Eng. 435–6, 12–18. (10.1016/j.msea.2006.07.020)

[7] Porter DL Evans AG Heuer AH 1979 Transformation toughening in partially stabilized zirconia (PSZ). Acta Metall. 27, 1649–1654. (10.1016/0001-6160(79)90046-4)

[8] Newman JC 1981 A crack-closure model for predicting crack growth under aircraft spectrum loading. In Methods and models for predicting fatigue crack growth under random loading (eds Chang JB Hudson CM), pp. 53–84. STP748.West Conshohocken, PA: ASTM.

[9] Clegg WJ 1992 The fabrication and failure of laminar ceramic composites. Acta Metall. Mater. 40, 3085–3093. (10.1016/0956-7151(92)90471-P)

[10] Hung Y-C Withers PJ 2012 Fibre bridging during high temperature fatigue crack growth in Ti/SiC composites. Acta Mater. 60, 958–971. (10.1016/j.actamat.2011.10.036)

[11] Withers PJ 2011 3D crack-tip microscopy: illuminating micro-scale effects on crack-tip behavior. Adv. Eng. Mater. 13, 1096–1100. (10.1002/adem.201100092)

[12] Withers PJ In Practical residual stress measurement methods (ed. Schajer GS), pp. 149–176. London, UK: John Wiley.

[13] Stock SR 2009 Microcomputed tomography: methodology and applications Boca Raton, FL: CRC Press.

[14] Banhart J 2008 Advanced tomographic methods in materials research and engineering Oxford, UK: Oxford University Press.

[15] Allison JE 1978 The measurement of crack tip stresses by X-ray diffraction. Technical report AFFDL-TR-78-24. Air Force Flight Dynamics Laboratory, Wright-Patterson Air Force Base, Ohio, USA

[16] Allen AJ Hutchings MT Windsor CG Andreani C 1985 Neutron diffraction methods for the study of residual stress fields. Adv. Phys. 34, 445–473. (10.1080/00018738500101791)

[17] Hutchings MT Hippsley CA Rainey V 1990 Neutron diffraction measurement of the stress field during fatigue cycling of a cracked test specimen. Mater. Res. Soc. Symp. Proc. 166, 317 (10.1557/PROC-166-317)

[18] Lee SY Liaw PK Choo H Rogge RB 2011 A study on fatigue crack growth behavior subjected to a single tensile overload. Part I. An overload-induced transient crack growth micromechanism. Acta Mater. 59, 485–494. (10.1016/j.actamat.2010.09.049)

[19] Lee SY Choo H Liaw PK An K Hubbard CR 2011 A study on fatigue crack growth behavior subjected to a single tensile overload. Part II. Transfer of stress concentration and its role in overload-induced transient crack growth. Acta Mater. 59, 495–502. (10.1016/j.actamat.2010.09.048)

[20] Steuwer A Santisteban JR Turski M Withers PJ Buslaps T 2005 High-resolution strain mapping in bulk samples using full-profile analysis of energy dispersive synchrotron X-ray diffraction data. Nucl. Instrum. Methods Phys. Res. B 238, 200–204. (10.1016/j.nimb.2005.06.049)

[21] Croft M 2005 Strain profiling of fatigue crack overload effects using energy dispersive X-ray diffraction. Int. J. Fatigue 27, 1408–1419. (10.1016/j.ijfatigue.2005.06.022)

[22] Steuwer A Rahman M Shterenlikht A Fitzpatrick ME Edwards L Withers PJ 2010 The evolution of crack-tip stresses during a fatigue overload event. Acta Mater. 58, 4039–4052. (10.1016/j.actamat.2010.03.013)

[23] Garvie RC Hannick RH Pascoe RT 1975 Ceramic steel??. Nature 258, 703–704. (10.1038/258703a0)

[24] Basu B 2005 Toughening of yttria-stabilised tetragonal zirconia ceramics. Int. Mater. Rev. 50, 239–256. (10.1179/174328005X41113)

[25] Hannink RHJ Kelly PM Muddle BC 2000 Transformation toughening in zirconia-containing ceramics. J. Am. Ceram. Soc. 83, 461–487. (10.1111/j.1151-2916.2000.tb01221.x)

[26] Meschke F Raddatz O Kolleck A Schneider GA 2000 R-curve behavior and crack-closure stresses in barium titanate and (Mg,Y)-PSZ ceramics. J. Am. Ceram. Soc. 83, 353–361. (10.1111/j.1151-2916.2000.tb01197.x)

[27] Ma WM Xiu ZM Bi XG Wen L Sun XD 2005 Phase transformation and machnical properties of Al_2_O_3_/ ZrO_2_(Y_2_O_3_) composites during fracturing. Acta Metall. Sin. 41, 93–98.

[28] Gollerthan S Young ML Baruj A Frenzel J Schmahl WW Eggeler G 2009 Fracture mechanics and microstructure in NiTi shape memory alloys. Acta Mater. 57, 1015–1025. (10.1016/j.actamat.2008.10.055)

[29] Hirose Y Sasaki T 1992 X-ray fractography. In Industrial applications of X-ray diffraction (eds Chung FH Smith DK), pp. 317–372. New York, NY: Marcel Dekker.

[30] Saprykin YV 1983 X-ray fractography of fatigue fractures in the zone of subcritical crack-growth. Strength Mater. 15, 1305–1312. (10.1007/bf01531847)

[31] Saprykin YV Kozlov PM Botvina LR Klevtsov GV 1978 A method of collimating-local examination of the zones of plastic deformation below the fracture surface. Fiz. Prochn. Plastichn. Met. Splavov. 1, 37–39.

[32] Dias A Lebrun JL Bignonnet A 1999 X-ray diffraction studies on fatigue crack plastic zones developed under plane strain state conditions. Fatigue Fract. Eng. Mater. Struct. 22, 133–144. (10.1046/j.1460-2695.1999.00142.x)

[33] Akita K Yoshioka Y Suzuki H Sasaki T 2000 X-ray fractography using synchrotron radiation: residual stress distribution just beneath fatigue fracture surface. Mater. Sci. Res. Int. 6, 269–274.

[34] Lopez-Crespo P Withers PJ Yates JR Steuwer A Buslaps T Tai YH In press. 10 (10.1046/j.2012.01670.x)

[35] Irwin GR 1960 Plastic zone near a crack and fracture toughness. In Proc. 7th Sagamore Ordinance Materials Conf., Raquette Lake, NY, 16–19 August 1960 New York: Syracruse University, pp. 63–78.

[36] Lopez-Crespo P Withers PJ Steuwer A Buslaps T Tai YH Lopez-Moreno A Yates JR 2015 Measuring overload effects during fatigue crack growth in bainitic steel by synchrotron X-ray diffraction. Int. J. Fract. 71, 11–16.

[37] Rajanna K Pathiraj B Kolster BH 1997 Duplex stainless steel fracture surface analysis using x-ray fractography. J. Mater. Eng. Perform. 6, 35–40. (10.1007/s11665-997-0029-9)

[38] Rajanna K Pathiraj B Kolster BH 1996 X-ray fractography studies on austenitic stainless steels. Eng. Fract. Mech. 54, 155 (10.1016/0013-7944(95)00246-4)

[39] Elber W 1970 Fatigue crack closure under cyclic tension. Eng. Fract. Mech. 2, 37–45. (10.1016/0013-7944(70)90028-7)

[40] Vasudevan AK Sadanada K Glinka G 2001 Critical parameters for fatigue. Int. J. Fatigue 23, 39 (10.1016/S0142-1123(01)00171-2)

[41] Sadananda K Vasudevan AK Holtz RL Lee EU 1999 Analysis of overload effects and related phenomena. Int. J. Fatigue 21, 233 (10.1016/S0142-1123(99)00094-8)

[42] Suresh S Zamiski GF Ritchie RO 1981 Oxide-induced crack closure: an explanation for near threshold corrosion fatigue crack growth behavior. Metall. Mater. Trans. A 12, 1435–1443. (10.1007/BF02643688)

[43] Suresh S 1991 Fatigue of materials. Cambridge, UK: Cambridge University Press.

[44] Alizadeh H Hills DA de Matos PFP Nowell D Pavier MJ Paynter RJ Smith DJ Simandjuntak S 2007 A comparison of two and three-dimensional analyses of fatigue crack closure. Int. J. Fatigue 29, 222–231. (10.1016/j.ijfatigue.2006.03.014)

[45] Fleck NA 1986 Finite-element analysis of plasticity-induced crack closure under plane-strain conditions. Eng. Fract. Mech. 25, 441–449. (10.1016/0013-7944(86)90258-4)

[46] Xu YG Gregson PJ Sinclair I 2000 Systematic assessment and validation of compliance-based crack closure measurements in fatigue. Mater. Sci. Eng. A 284, 114–125. (10.1016/s0921-5093(00)00758-9)

[47] Nowell D de Matos PFP Application of digital image correlation to the investigation of crack closure following overloads In Proc. 10th Int. Fatigue Congress (Fatigue 2010) Prague, Czech Republic, 6–11 June 2010 (ed. Lukáš P), pp. 1035–1043. Prague, Czech Republic: Academy of Sciences of the Czech Republic.

[48] Lopez-Crespo P Withers PJ Yusof F Dai H Steuwer A Kelleher JF Buslaps T 2013 Overload effects on fatigue crack-tip fields under plane stress conditions: surface and bulk analysis. Fatigue Fract. Eng. Mater. Struct 36, 75–84. (10.1111/j.1460-2695.2012.01670.x)

[49] Withers PJ Lopez-Crespo P Kyrieleis A Hung Y-C Insights into fracture mechanics for a Ti/SiC fibre composite by 4D X-ray tomography. In Proc. 31st Risø Int. Symp. Challenges in Materials Science and Possibilities in 3D and 4D Characterisation Techniques Risø, Denmark, 6–10 September 2010 (eds Hansen N Jensen DJ Nielsen SF Poulsen HF Ralph B), pp. 219–236. Roskilde, Denmark: Danmarks Tekniske Universitet, Risø Nationallaboratoriet for Bæredygtig Energi.

[50] Withers PJ Lopez-Crespo P Kyrieleis A Hung Y-C 2012 Evolution of crack-bridging and crack-tip driving force during the growth of a fatigue crack in a Ti/SiC composite. Proc. R. Soc. A 468, 2722–2743. (10.1098/rspa.2012.0070)

[51] Stock SR 1999 X-ray microtomography of materials. Int. Mater. Rev. 44, 141–164. (10.1179/095066099101528261)

[52] Breunig TM Stock SR Guvenilir A Elliott JC Anderson P Davis GR 1993 Damage in aligned-fibre SiC/Al quantified using a laboratory X-ray tomographic microscope. Composites 24, 209–214. (10.1016/0010-4361(93)90165-5)

[53] Schilling PJ Karedla BPR Tatiparthi AK Verges MA Herrington PD 2005 X-ray computed microtomography of internal damage in fiber reinforced polymer matrix composites. Compos. Sci. Technol. 65, 2071–2078. (10.1016/j.compscitech.2005.05.014)

[54] Scott AE Mavrogordato M Wright P Sinclair I Spearing SM 2011 In situ fibre fracture measurement in carbon-epoxy laminates using high resolution computed tomography. Compos. Sci. Technol. 71, 1471–1477. (10.1016/j.compscitech.2011.06.004)

[55] Withers PJ Bennett J Hung Y-C Preuss M 2006 Crack opening displacements during fatigue crack growth in Ti-SiC fibre metal matrix composites by X-ray tomography. Mater. Sci. Technol. 22, 1052–1058. (10.1179/174328406X114108)

[56] Hung Y-C 2009 Investigation of fatigue crack bridging in Ti/SiC composites by X-ray microtomography and diffraction. PhD thesis, University of Manchester, UK

[57] Limodin N Rethore J Buffiere JY Hild F Roux S Ludwig W Rannou J Gravouil A 2010 Influence of closure on the 3D propagation of fatigue cracks in a nodular cast iron investigated by X-ray tomography and 3D volume correlation. Acta Mater. 58, 2957–2967. (10.1016/j.actamat.2010.01.024)

[58] Buffiere JY Ferrie E Proudhon H Ludwig W 2006 Three-dimensional visualisation of fatigue cracks in metals using high resolution synchrotron X-ray micro-tomography. Mater. Sci. Technol. 22, 1019–1024. (10.1179/174328406x114135)

[59] Toda H Sinclair I Buffiere JY Maire E Connolley T Joyce M Khor KH Gregson P 2003 Assessment of the fatigue crack closure phenomenon in damage-tolerant aluminium alloy by in-situ high-resolution synchrotron X-ray microtomography. Philos. Mag. 83, 2429–2448. (10.1080/147864303100015754)

[60] Braz D da Motta LMG Lopes RT 1999 Computed tomography in the fatigue test analysis of an asphaltic mixture. Appl. Radiat. Isotopes 50, 661–671. (10.1016/s0969-8043(98)00122-5) 11003512

[61] Stock SR Ignatiev K Davis GR Elliott JC Fezzaa K Lee W-K 2002 Fatigue cracks in aluminum samples studied with X-ray phase contrast imaging and with absorption microstomography. In Advances in X-ray analysis 123–127. New York, NY:JCPDS-International Centre for Diffraction Data.

[62] Birosca S Buffiere JY Karadge M Preuss M 2011 3-D observations of short fatigue crack interaction with lamellar and duplex microstructures in a two-phase titanium alloy. Acta Mater. 59, 1510–1522. (10.1016/j.actamat.2010.11.015)

[63] Marrow TJ Buffiere JY Withers PJ Johnson G Engelberg D 2004 High resolution X-ray tomography of short fatigue crack nucleation in austempered ductile cast iron. Int. J. Fatigue 26, 717–725. (10.1016/j.ijfatigue.2003.11.001)

[64] Tsuritani H Sayama T Okamoto Y Takayanagi T Uesugi K Mori T 2011 Application of synchrotron radiation X-ray microtomography to nondestructive evaluation of thermal fatigue process in flip chip interconnects. J. Electron. Packag. 133, 021007 (10.1115/1.4003992)

[65] Cloetens P Pateyron-Salome M Buffiere JY Peix G Baruchel J Peyrin F Schlenker M 1997 Observation of microstructure and damage in materials by phase sensitive radiography and tomography. J. Appl. Phys. 81, 5878–5886. (10.1063/1.364374)

[66] Buffiere JY Savelli S Jouneau PH Maire E Fougeres R 2001 Experimental study of porosity and its relation to fatigue mechanisms of model Al-Si7-Mg0.3 cast Al alloys. Mater. Sci. Eng. 316, 115–126. (10.1016/S0921-5093(01)01225-4)

[67] Ludwig W Buffiere JY Savelli S Cloetens P 2003 Study of the interaction of a short fatigue crack with grain boundaries in a cast Al alloy using X-ray microtomography. Acta Mater. 51, 585–598. (10.1016/s1359-6454(02)00320-8)

[68] Khor KH Buffiere JY Ludwig W Toda H Ubhi HS Gregson PJ Sinclair I 2004 In situ high resolution synchrotron x-ray tomography of fatigue crack closure micromechanisms. J. Phys. Condensed Matter 16, 3511 (10.1088/0953-8984/16/33/012)

[69] Khor KH Buffiere JY Ludwig W Sinclair I 2006 High resolution X-ray tomography of micromechanisms of fatigue crack closure. Scr. Mater. 55, 47–50. (10.1016/j.scriptamat.2006.01.016)

[70] King A Johnson G Engelberg D Ludwig W Marrow J 2008 Observations of intergranular stress corrosion cracking in a grain-mapped polycrystal. Science 321, 382–385. (10.1126/science.1156211) 18635797

[71] Fu X Poulsen HF Schmidt S Nielsen SF Lauridsen EM Jensen DJ 2003 Non-destructive mapping of grains in three dimensions. Scr. Mater. 49, 1093–1096. (10.1016/j.scriptamat.2003.08.007)

[72] King A Ludwig W Herbig M Buffiere JY Khan AA Stevens N Marrow TJ 2011 Three-dimensional in situ observations of short fatigue crack growth in magnesium. Acta Mater. 59, 6761–6771. (10.1016/j.actamat.2011.07.034)

[73] Ritchie RO 1988 Mechanisms of fatigue crack propagation in metals, ceramics and composites: role of crack tip shielding. Mater. Sci. Eng. A 103, 15–28. (10.1016/0025-5416(88)90547-2)

[74] Stock SR Mesotexture, deflection and closure of fatigue cracks in Ai-Li 2090 T8E41. In Advanced materials for the 21st century: the 1999 Julia R Weertman Symposium (eds Chung YW *et al*), pp. 251–258. Warrendale, PA: The Minerals & Metals Society.

[75] Zimmerman MH Baskin DM Faber KT Fuller ER Allen AJ Keane DT 2001 Fracture of a textured anisotropic ceramic. Acta Mater. 49, 3231–3242. (10.1016/s1359-6454(01)00224-5)

[76] Zimmermann EA 2011 Age-related changes in the plasticity and toughness of human cortical bone at multiple length scales. Proc. Natl Acad. Sci. USA 108, 14416–14421. (10.1073/pnas.1107966108) 21873221 PMC3167515

[77] Stock SR Vieira AEM Delbem ACB Cannon ML Xiao X de Carlo F 2008 Synchrotron microcomputed tomography of the mature bovine dentinoenamel junction. J. Struct. Biol. 161, 162–171. (10.1016/j.jsb.2007.10.006) 18054250

[78] Kruzic J Nalla RK Kinney JH Ritchie RO 2003 Crack blunting, crack bridging and resistance-curve fracture mechanics in dentin: effect of hydration. Biomaterials 24, 5209–5221. (10.1016/s0142-9612(03)00458-7) 14568438

[79] Leng H Wang X Ross RD Niebur GL Roeder RK 2008 Micro-computed tomography of fatigue microdamage in cortical bone using a barium sulfate contrast agent. J. Mech. Behav. Biomed. Mater. 1, 68–75. (10.1016/j.jmbbm.2007.06.002) 18443659 PMC2352164

[80] Koester KJ Ager JW Ritchie RO 2008 The true toughness of human cortical bone measured with realistically short cracks. Nat. Mater. 7, 672–677. (10.1038/nmat2221) 18587403

[81] Clegg WJ Kendall K Alford NM Birchall D Button TW 1990 A simple way to make tough ceramics. Nature 347, 455–457. (10.1038/347455a0)

[82] Roy S Butz B Wanner A 2010 Damage evolution and domain-level anisotropy in metal/ceramic composites exhibiting lamellar microstructures. Acta Mater. 58, 2300–2312. (10.1016/j.actamat.2009.12.015)

[83] Vashishth D Behiri JC Bonfield W 1997 Crack growth resistance in cortical bone: concept of microcrack toughening. J. Biomech. 30, 763–769. (10.1016/s0021-9290(97)00029-8) 9239560

[84] Larrue A Rattner A Peter Z-A Olivier C Laroche N Vico L Peyrin F 2011 Synchrotron radiation micro-CT at the micrometer scale for the analysis of the three-dimensional morphology of microcracks in human trabecular bone. PLoS ONE 6, 21297 (10.1371/journal.pone.0021297) PMC3131277 21750707

[85] Nalla RK Altenberger I Noster U Liu GY Scholtes B Ritchie RO 2003 On the influence of mechanical surface treatments—deep rolling and laser shock peening—on the fatigue behaviour of Ti–6Al–4V at ambient and elevated temperatures. Mater. Sci. Technol. A 355, 216–230. (10.1016/S0921-5093(03)00069-8)

[86] Voide R Schneider P Stauber M Wyss R Stampanoni M Sennhauser U van Lenthe GH Mueller R 2009 Time-lapsed assessment of microcrack initiation and propagation in murine cortical bone at submicrometer resolution. Bone 45, 164–173. (10.1016/j.bone.2009.04.248) 19410668

[87] Christen D Levchuk A Schori S Schneider P Boyd SK Muller R 2012 Deformable image registration and 3D strain mapping for the quantitative assessment of cortical bone microdamage. J. Mech. Behav. Biomed. Mater. 8, 184–193. (10.1016/j.jmbbm.2011.12.009) 22402165

[88] Nalla RK Kruzic JJ Ritchie RO 2004 On the origin of the toughness of mineralized tissue: microcracking or crack bridging??. Bone 34, 790–798. (10.1016/j.bone.2004.02.001) 15121010

[89] Nalla RK Kinney JH Ritchie RO 2003 Effect of orientation on the in vitro fracture toughness of dentin: the role of toughening mechanisms. Biomaterials 24, 3955–3968. (10.1016/s0142-9612(03)00278-3) 12834591

[90] Launey ME Buehler MJ Ritchie RO 2010 On the mechanistic origins of toughness in bone. Annu. Rev. Mater. Res. 40, 25–53. (10.1146/annurev-matsci-070909-104427)

[91] Guvenilir A Breunig TM Kinney JH Stock SR 1997 Direct observations of crack opening as a function of applied load in the interior of a notched tensile sample of Al–Li 2090. Acta Mater. 45, 1977–1987. (10.1016/S1359-6454(96)00311-4)

[92] Guvenilir A Stock SR 1998 High resolution computed tomography and implications for fatigue crack closure modelling. Fatigue Fract. Eng. Mater. Struct. 21, 439–450. (10.1046/j.1460-2695.1998.00062.x)

[93] Guvenilir A Breunig TM Kinney JH Stock SR 1999 New direct observations of crack closure in Al–Li 2090 T8E41. Phil. Trans. R. Soc. Lond. A 357, 2755–2775. (10.1098/rsta.1999.0464)

[94] Ignatiev KI Davis GR Elliott JC Stock SR 2006 MicroCT (microtomography) quantification of microstructure related to macroscopic behaviour. Part 1: fatigue crack closure measured in situ in AA 2090 compact tension samples. Mater. Sci. Technol. 22, 1025–1037. (10.1179/174328406x114144)

[95] Limodin N Rethore J Buffiere JY Gravouil A Hild F Roux S 2009 Crack closure and stress intensity factor measurements in nodular graphite cast iron using three-dimensional correlation of laboratory X-ray microtomography images. Acta Mater. 57, 4090–4101. (10.1016/j.actamat.2009.05.005)

[96] Zhang H Toda H Qu PC Sakaguchi Y Kobayashi M Uesugi K Suzuki Y 2009 Three-dimensional fatigue crack growth behavior in an aluminum alloy investigated with in situ high-resolution synchrotron X-ray microtomography. Acta Mater. 57, 3287–3300. (10.1016/j.actamat.2009.03.036)

[97] Zhu X-K Joyce JA 2012 Review of fracture toughness (G, K, J, CTOD, CTOA) testing and standardization. Eng. Fract. Mech. 85, 1–46. (10.1016/j.engfracmech.2012.02.001)

[98] Becker TH Mostafavi M Tait RB Marrow TJ 2012 An approach to calculate the J-integral by digital image correlation displacement field measurement. Fatigue Fract. Eng. Mater. Struct. 35, 971–984. (10.1111/j.1460-2695.2012.01685.x)

[99] Buchanan DJ John R Johnson DA 1997 Determination of crack bridging stresses from crack opening displacments. Int. J. Fract. 87, 101–117. (10.1023/A:1007495331890)

[100] Bueckner HF 1970 A novel principle for computation of stress intensity factors. Z. Angew. Math. Mech. 50, 529–546.

[101] Ghosn LJ Kantzos P Telesman J 1992 Modeling of crack bridging in a unidirectional metal matrix composite. Int. J. Fract. 54, 345–357. (10.1007/bf00035108)

[102] Toda H Sinclair I Buffiere JY Maire E Khor KH Gregson P Kobayashi T 2004 A 3D measurement procedure for internal local crack driving forces via synchrotron X-ray microtomography. Acta Mater. 52, 1305–1317. (10.1016/j.actamat.2003.11.014)

[103] Rannou J 2010 Three dimensional experimental and numerical multiscale analysis of a fatigue crack. Comput. Meth. Appl. Mech. Eng. 199, 1307–1325. (10.1016/j.cma.2009.09.013)

[104] Scheuerlein C Di Michiel M Buta F 2009 Synchrotron radiation techniques for the characterization of Nb3Sn superconductors. IEEE Trans. Appl. Supercond. 19, 2653–2656. (10.1109/tasc.2009.2019101)

[105] Rossoll A Moser B Mortensen A 2012 Tensile strength of axially loaded unidirectional Nextel 610 (TM) reinforced aluminium: a case study in local load sharing between randomly distributed fibres. Compos. A, Appl. Sci. Manuf. 43, 129–137. (10.1016/j.compositesa.2011.09.027)

[106] Turski M Bouchard PJ Steuwer A Withers PJ 2008 Residual stress driven creep cracking in type 316 stainless steel. Acta Mater. 56, 3598–3612. (10.1016/j.actamat.2008.03.045)

[107] Bouchard PJ Withers PJ MacDonald S Hennan R 2004 Quantification of creep cavitation damage mapping around a crack in a stainless steel pressure vessel. Acta Mater. 52, 23–34. (10.1016/j.actamat.2003.08.022)

[108] Furuta A Uchino K 1993 Dynamic observation of crack-propagation in piezoelectric multilayer actuators. J. Am. Ceram. Soc. 76, 1615–1617. (10.1111/j.1151-2916.1993.tb03950.x)

[109] Hall DA Steuwer A Cherdhirunkorn B Withers PJ Mori T 2005 Micromechanics of residual stress and texture development due to poling in polycrystalline ferroelectric ceramics. J. Mech. Phys. Sol. 53, 249–260. (10.1016/j.jmps.2004.07.002)

[110] Eggeler G Hornbogen E Yawny A Heckmann A Wagner M 2004 Structural and functional fatigue of NiTi shape memory alloys. Mater. Sci. Eng. A 378, 24–33. (10.1016/j.msea.2003.10.327)

[111] McKelvey AL Ritchie RO 2001 Fatigue-crack growth behavior in the superelastic and shape-memory alloy nitinol. Metall. Mater. Trans. A 32, 731–743. (10.1007/s11661-001-1008-7)

[112] Song GM Pei YT Sloof WG Li SB de Hosson JTM Van der Zwaag S 2008 Oxidation-induced crack healing in Ti_3_AlC_2_ ceramics. Scr. Mater. 58, 13–16. (10.1016/j.scriptamat.2007.09.006)

[113] Di Michiel M Merino JM Fernandez-Carreiras D Buslaps T Honkimaki V Falus P Martins T Svensson O 2005 Fast microtomography using high energy synchrotron radiation. Rev. Sci. Instrum. 76, 043702 (10.1063/1.1884194)

[114] Maire E Withers PJ 2014 Quantitative X-ray tomography. Int. Mater. Rev. 59, 1–43. (10.1179/1743280413Y.0000000023)

[115] Burnett TL 2014 Correlative tomography. Sci. Rep. 4, 4711 (10.1038/srep04711) 24736640 PMC3988479

